# Recombinant L-asparaginase from *Stenotrophomonas maltophilia*: a promising low-immunogenic anticancer agent

**DOI:** 10.1186/s12934-025-02856-0

**Published:** 2025-12-19

**Authors:** Nada A. Abdelrazek, Sarra E. Saleh, Amal E. Ali, Mohammad M. Aboulwafa, Marwa M. Raafat

**Affiliations:** 1 Department of Microbiology and Immunology, Faculty of Pharmacy, Future University in Egypt, Cairo, Egypt; 2 Department of Microbiology and immunology, Faculty of Pharmacy, Ain Shams University, Al Khalifa Al Maamoun St., Abbassia, Cairo, 11517 Egypt; 3 Department of Microbiology and Immunology, Faculty of Pharmacy, Cairo University, 11562 Cairo, Egypt; 4 Department of Microbiology and Immunology, Faculty of Pharmacy, King Salman International University, South Sinai, Ras-Sudr, Egypt

**Keywords:** *Stenotrophomonas maltophilia*, L-asparaginase, Cloning, Expression, Cytotoxicity, Immunogenicity

## Abstract

L-asparaginase is a crucial enzyme used in chemotherapy regimens for the treatment of acute lymphoblastic leukemia (ALL), its incorporation in the pediatric treatment protocols helped in achieving a high cure rate. However, immunogenic side-effects restrict its application and frequently result in stopping treatment. There is a current need for the identification of novel L-asparaginase with improved properties and lower adverse effects compared to those available in the market. L-asparaginase gene from *Stenotrophomonas maltophilia* (*S. maltophilia*), an isolated organism that was mentioned as a novel and excellent source for L- asparaginase, was cloned and expressed using *E. coli* DH5α and *E. coli* BL21(DE3). Investigations of different conditions of expression of recombinant L-asparaginase in *E. coli* BL21(DE3) using Box–Behnken design predicted maximum expression at 37 °C temperature, 250 rpm agitation, 0.83 mM isopropylthio-β-D-galactoside (IPTG) concentration after incubation for 17 h. The optimized expression conditions were validated using L-asparaginase activity assay. The obtained recombinant protein was purified using Ni-NTA spin column. SDS-PAGE demonstrated a single band of 17 KDa apparent molecular weight. The kinetic parameters were determined, and they exhibited a low Km value of 2.94 × 10^− 2^ M and Vmax of 14.73 IU/ml. Cytotoxicity on various cell lines was tested in relation to marketed *E. coli* L-asparaginase and exhibited low IC50 of 1.92 IU/ml and 2.03 IU/ml for HEP-G2 and K-562 cell lines, respectively. Additionally, mice treated with recombinant L-asparaginase displayed a significantly lower immunological response (IgG) compared to mice treated with marketed *E. coli* L-asparaginase (*p*-value < 0.0001). These findings demonstrate the potentiality of recombinant L-asparaginase for its development as a chemotherapeutic drug.

## Introduction

Enzymes function as biocatalysts, accelerating chemical reactions to produce valuable products at the end of the process [1]. Nowadays, Enzymes hold a prominent position in the pharmaceutical sector. This is because of their exceptional efficiency and selectivity as catalysts for biological activities. Most of these therapeutic enzymes could be commercially available with the aid of different fermentation processes and appropriate expression systems, such as microbial strains (e.g. yeast, bacteria, or fungi), animal or plant cell cultures, and genetically modified species. The improvement in the recombinant DNA technology, material science, protein engineering, nanotechnology, and enzyme immobilization, have been formed models for the creation of enzymatic drugs with a wide range of therapeutic applications [2].

ALL is a form of cancer where the lymphoid parent cells rapidly multiply and convert to malignant cells. ALL is the most prevalent kind of cancer diagnosed in children, accounting for about 25% of all cancer diagnoses [3]. Previously, vincristine and prednisone were part of the standard chemotherapy regimen for the management of ALL. The addition of L-asparaginase to the treatment protocol had improved the clinical results in comparison to the usage of vincristine and prednisone only [4]. L-asparaginase is an amidohydrolase that catalyzes the breakdown of L-asparagine into aspartic acid and ammonia. Asparagine synthetase, an enzyme that ordinarily exists in normal cells and is responsible for synthesising L-asparagine, is absent from leukemic cells, which explains the particular activity of L-asparaginase on leukemic cells [5]. Thereby, they make use of the extracellular supply of L-asparagine for their malignant proliferation. Consequently, the exogenous L-asparagine was depleted by L-asparaginase, leading to nutritional stress and ultimately leukemic cell apoptosis and death [6].

Birds, plants, mammals including guinea pigs and rodents, and microorganisms like bacteria, fungi and yeasts are all common sources of L-asparaginase [7]. Because microorganisms are generally easier to grow and culture in large quantities, they have emerged as a preferred agent for producing proteins, enzymes, and other target molecules. Additionally, microbial enzymes often have a wider range of characteristics and are more stable than those derived from plants and animals [8].

The enzyme L-asparaginase, either from *E. coli* (Elspar^®^) or *Erwinia chrysanthemi * (Erwinaze^®^), have been approved by FDA for the treatment of ALL. The use of enzyme is associated with different side effects as hypersensitivity, hepatotoxicity, coagulation disorder, pancreatitis, etc. due to the formation of antibodies [7]. So, the development of an alternative L-asparaginase with high therapeutic efficiency and minimum adverse effects is highly needed to address this challenge.

*S. maltophilia* is a gram negative microorganism, it can be isolated from water, soil, and plants [9]. Several studies focused on the use of *S. maltophilia* in various biotechnological applications. This bacterium can convert a variety of substrates into products of significant environmental and technological importance [10]. *S. maltophilia* has the ability to produce different extracellular enzymes such as lipase, gelatinase, keratinase, and L-asparaginase.

In the light of this, The main focus of the current investigation was to determine whether L-asparaginase from the novel microbe *S. maltophilia* EMCC2297, can be employed as a safe and effective chemotherapy agent. Therefore, the gene coded for L-asparaginase from the soil isolate *S. maltophilia* EMCC2297 was cloned and heterologously expressed in *E. coli* strain. The expressed L-asparaginase was optimized for maximum expression, tested for antitumor activity in vitro and assessed for its ability to induce the immune response using in vivo animals.

## Materials and methods

### Bacterial isolate

The *S. maltophilia* soil isolate was found through a comprehensive screening program comprising 722 soil isolates to be a potential candidate for the production of L-asparaginase. The 16 S rRNA sequence of this isolate was submitted to the NCBI database under Accession code MG66599, and it was placed in the Microbiological Resources Center (Cairo Mircen) under the code EMCC2297 [11]. A single pure colony were monthly sub-cultured on slants of Luria Bertani (LB) agar, (Himedia, India) for short-term preservation, and the isolate was stored as stock in 50% glycerol at -80 °C for long-term preservation.

### Cloning and heterologous expression of *S. maltophilia* L-asparaginase gene

#### Isolation of DNA and polymerase chain reaction (PCR) amplification of L-asparaginase gene

The genomic DNA of *S. maltophilia* EMCC2297 was extracted using QIAMP mini kit (Qiagen^®^, Germany) according to the protocol supplemented by the manufacturer. The competent cells (*E. coli*) were prepared and transformed, and the DNA was manipulated in vitro in accordance with the conventional techniques [12]. The extracted genomic DNA was utilized as a template to amplify the gene coding for L-asparaginase by PCR. Using *S. maltophilia* NCTC13014 sequence from the GenBank. The query primers were designed with the aid of OligoAnalyzer 3.1 tool (Integrated DNA Technologies, Inc.) (NA001-F & NA002-R). L-asparaginase type II NA001-F (forward 5’-CGCCATATGATGCACATGATGGAAGAG-3’) and NA002-R (reverse 5’-CGGGATCCTCAVACC GAYTCGAA-3’) were the oligonucleotide sequences used.

The primers were modified to include *Bam*HI and *Nde*I (Thermo Fischer, USA) restriction sites at their respective 5’ ends to aid in the directed cloning of the structural L-asparaginase II gene. L-asparaginase gene was amplified using the designed primers; the PCR was conducted using T100 thermal cycler (BioRad^®^) and the mixture of the PCR (50 µl) was constructed according to the protocol provided with Phusion high-fidelity PCR master mix (Thermo Fisher Scientific, USA) using 0.5 µM of each primer and 200 ng genomic DNA. The PCR was carried out according to the following conditions: initial denaturation at 98 °C for 3 min after that a number of 35 cycles of denaturation at 98 °C for 10 s, annealing at 55 °C for 30 s and extension at 72 °C for 30 s. Final extension was performed at 72 °C for 10 min. The resultant amplicon was visualized utilizing agarose gel electrophoresis [12].

#### Recombinant plasmid construction for cloning and expression

The PCR products were doubly digested by *Bam*HI and *Nde*I (Thermo Fischer, USA), at the same time pET-22b (+) plasmid (Novagen, Merck, CA, USA) was identically processed. The doubly digested plasmid and gene were ligated by the aid of T4 DNA ligase (Takara, Japan). According to the modified Hanahan method, the recombinant plasmid pET-22b(+)-ASP was introduced into previously chemically prepared competent *E. coli* DH5α. Transformed cells were then inoculated on plates of LB agar supplemented by 100 µg/ml ampicillin [13]. Plates of LB/ampicillin agar were kept at 37 °C for 18 h. Colony PCR was used to investigate the obtained colonies using the gene specific primers (NA001-F and NA002-R) to screen the existence of the gene of interest in the produced clones [14–16]. Random colonies were carefully picked from the overnight grown plates using sterile toothpick and were mixed with sterile water aliquots of volume 50 µL. Prior to initiating the colony PCR, the mixture was heated for 5 min to 95 °C [17]. All the obtained PCR products were tested using 1.2% agarose gel electrophoresis. The successful clone (bearing the insert) was used for the propagation of the recombinant plasmid. Using QIAprep spin miniprep kit (Qiagen^®^, Germany), the recombinant plasmids was extracted and transformed using heat shock technique into chemically prepared competent *E. coli* BL21 (DE3) to produce *E. coli* BL21 (DE3)-pET-22b(+)-ASP [18] .

#### Heterologous expression of L-asparaginase gene in *E. coli* BL21 (DE3)

L-asparaginase expression was conducted according to the procedures described by Studier *et al.*, (1990). Under the control of T7 promoter the expression was carried utilizing *E. coli* BL21 (DE3) to be a host strain. One colony of *E. coli* BL21 (DE3)-pET-22b(+)-ASP bearing the recombinant plasmid (pET-22b - L-asparaginase) and another colony with an empty plasmid (as a control to confirm the efficacy of transformation) were incubated overnight in 3 ml aliquots LB broth supplemented with ampicillin (100 µg/ml) at 37 °C, 250 rpm for 16 h in an incubator shaker to prepare a pre-culture. The overnight developed pre-culture was added to fresh LB/ampicillin broth at a ratio of 1:100 v/v, and the mixture was then incubated at 37 °C/250 rpm until the midlogarithmic phase was achieved (OD_600_ 0.5–0.6). The expression conditions were optimized by testing different factors using one factor at a time technique (OFAT) to achieve the highest yield of the recombinant L-asparaginase protein. To determine the results of the tested factors. The cell free lysate was prepared and quantitatively examined for L-asparaginase productivity.

### Studying the influence of different factors on the expression of L-asparaginase by the Recombinant *E. coli* BL21 (DE3)-pET-22b(+)-ASP

#### Incubation temperature

The expression of L-asparaginase by the recombinant *E. coli* BL21 (DE3) was assessed in response to a range of incubation temperatures (20, 25, 30, 37, and 40 °C). For these tests Erlenmeyer flasks containing LB/ampicillin broth were inoculated with the pre-culture to reach an OD_600_ of 0.5. IPTG (Sigma-Aldrich, USA) was added at 1 mM final concentration to induce the heterologous expression of L-asparaginase at 250 rpm of overnight incubation at the tested temperature. Cell free lysates were collected to test L-asparaginase productivity at the end of the incubation phase [19].

#### Incubation time after IPTG addition (Induction time)

The inoculated Erlenmeyer flasks containing LB/ampicillin broth were induced by 1 mM IPTG kept at 37 °C incubation temperature and 250 rpm shaking for different time intervals (0, 1, 2, 4, 16, 18, 20, 24 h). Cell free lysates of the tested organism were collected to test L-asparaginase productivity by the end of each incubation phase [19–21].

#### Effect of IPTG concentration

Various concentrations of IPTG (0, 0.2, 0.4, 0.5, 0.7, 0.8, 1, 1.2 mM) were tested to evaluate their effects on L-asparaginase expression. LB/ampicillin broth flasks containing the recombinant *E. coli* BL21 (DE3)-pET-22b(+)-ASP of OD_600_ 0.5 were induced by various concentrations of IPTG and kept at 37 °C and 250 rpm overnight. Cell free lysates were collected to test L-asparaginase productivity by the end of the incubation phase [21, 22].

#### Effect of agitation rate

Different agitation levels which included 150, 180, 200, 250 and 270 rpm were examined for their effects on the expression of L-asparaginase by the recombinant *E. coli* BL21 (DE3). The flasks containing the recombinant *E. coli* BL21 (DE3)-pET22b(+)-ASP were kept at 37 °C and 1 mM IPTG concentration overnight. Cell free lysates of the test organism were collected to test L-asparaginase productivities by the end of the incubation phase for the different tested agitation levels [22].

#### Optimization of L-asparaginase expression by the Recombinant *E. coli* BL21 (DE3) pET-22b(+)-ASP utilizing response surface methodology (RSM)

The Box-Behnken Central Composite design of RSM experimental design was used to optimize four process parameters [23] based on preliminary studies. The effect of incubation temperature coded (A), induction time coded (B), IPTG concentration coded (C), and agitation speed coded (D) were tested to study their interactions on L-asparaginase expression by the test organism. Each variable was tested at 3 levels that showed the maximum productivities of L-asparaginase, the mean level (coded 0) of each reflects one of maximum L-asparaginase expression while the other two levels denote the one above (coded + 1) and the one below (coded − 1) this mean level. The variables range that was examined is shown in Table 1. Statgraphics^®^ Centurion XV software, version 15.2.05 (StatPoint, Inc., Warrenton, VA, USA), was utilized for experimental design, graphical analysis of the data, and regression determination as well. Twenty-seven experiments were obtained and were adjusted at the values listed in Table 2. The experiments were performed in triplicates as previously stated except that the environmental parameters (incubation temperature, post-induction incubation time, IPTG concentration and agitation rate) were adjusted at the values indicated in Table S2. The program was utilized to analyze the data from the 27 completed tests in order to establish the optimum levels of the test variables, the pareto chart, the response surface contour plots, and the regression equation.

**Table 1:** Levels of reaction conditions of L-asparaginase expression process parameters as independent factors

Test variable	Variable code	Variables levels
		-1 0 +1
Incubation temperature (°C)	A	25 31 37
Incubation time (h)	B	16 20 24
IPTG concentration (mM)	C	0.2 0.6 1
Agitation (RPM)	D	180 215 250

**Table 2:** Experiments which were inferred by experimental design (RSM) and examined for the production of L-asparaginase by *E. coli* BL21 (DE3)

Exp.	A: Temperature (°C)		B: Time (h)		C: IPTG concentration (mM)		D: Agitation (rpm)	
	Value	Level code	Value	Level code	Value	Level code	Value	Level code
1	25	-1	20	0	0.2	-1	215	0
2	37	1	20	0	0.2	-1	215	0
3	31	0	16	-1	1	1	215	0
4	37	1	20	0	0.6	0	250	1
5	31	0	16	-1	0.2	-1	215	0
6	31	0	20	0	0.2	-1	180	-1
7	25	-1	20	0	0.6	0	180	-1
8	31	0	24	1	1	1	215	0
9	31	0	24	1	0.2	-1	215	0
10	37	1	20	0	0.6	0	180	-1
11	31	0	24	1	0.6	0	250	1
12	37	1	24	1	0.6	0	215	0
13	25	-1	20	0	1	1	215	0
14	25	-1	20	0	0.6	0	250	1
15	31	0	20	0	0.6	0	215	0
16	31	0	20	0	0.2	-1	250	1
17	31	0	20	0	0.6	0	215	0
18	25	-1	24	1	0.6	0	215	0
19	31	0	20	0	1	1	250	1
20	31	0	16	-1	0.6	0	180	-1
21	37	1	20	0	1	1	215	0
22	31	0	20	0	0.6	0	215	0
23	31	0	16	-1	0.6	0	250	1
24	37	1	16	-1	0.6	0	215	0
25	31	0	24	1	0.6	0	180	-1
26	31	0	20	0	1	1	180	-1
27	25	-1	16	-1	0.6	0	215	0

#### Validation of L-asparaginase expression by the Recombinant *E. coli* BL21 (DE3) utilizing the optimized conditions resulted by RSM

L-asparaginase productivity of the recombinant enzyme was assessed utilizing the optimized conditions obtained from the design used to validate the optimization of the RSM.

#### Purification of His tagged L-asparaginase produced by the Recombinant *E. coli* BL21 (DE3)

For the preparation of cell free lysate; 5 mL of the overnight culture of the test organism grown in LB/ampicillin was centrifuged at 12,000 rpm, and 4 °C for 5 min [24]. Then, the cells were washed twice with 25 mM Tris HCl buffer pH 7.5 at cold temperature [20]. The cells were resuspended in the lysis buffer (50 mM sodium phosphate buffer, 500 mM NaCl and 10 mM imidazole of pH 7.4) [25]. The cell suspension contained in a falcon tube was subsequently disrupted by intermittent sonication at 60 W for 2.5 min (15 s on followed by 30 s off) while the tube immersed in an ice bath. The cell debris in the sonicated suspension were precipitated by centrifugation using cooling centrifuge at 12,000 rpm, 4 °C for 30 min and the resulted lysate was then filtered using 0.22 μm sterile syringe filter [20]. To purify the 6x His tagged L-asparaginase protein, Ni-NTA spin columns (Qiagen, Germany) were used, and the procedures were followed according to the protocol provided by the manufacturer. The obtained purified protein was determined utilizing SDS and the protein concentration was detected using Bradford method [26].

#### L-asparaginase activity assay

L-asparaginase activity was tested using Mashburn and Wriston method [27]. The mixture of the reaction composed of 0.5 ml of asparagine (0.04 M), 0.05 ml of Tris-HCl buffer (pH 8.6), and 0.5 ml of purified enzyme. The reaction mixtures were incubated at 37 °C for 30 min. To stop the enzyme’s activity, 10% W/V TCA was applied. The combination was centrifuged for 5 min at 10,000 rpm. After that, 0.1 ml of the supernatant was mixed with 3.7 ml of distilled water, and 0.2 ml of Nessler’s reagent was then added. The resulting mixture was then let to stand at room temperature for 20 min. Using spectrophotometry, the absorbance was determined at 480 nm. The standard curve of the ammonium sulphate was used to determine the amount of ammonia released [28]. The release of one micromole of ammonia per hour at 37 °C and pH 8.6 is the definition of one unit of L-asparaginase activity [29, 30].

#### Assessment of the kinetic parameters of the recombinant L-asparaginase produced by the recombinant *E. coli* BL21 (DE3)-pET-22b(+)-ASP

Utilizing L-asparagine as a substrate, the kinetic parameters were measured at various concentrations between 0.01 and 0.12 M. These concentrations were applied in the reaction mixture of the L-asparaginase assay in 0.05 M Tris HCl buffer (pH 8.6) at 37 °C. The activity obtained at each concentration of L-asparagine was used to calculate the Michaelis constant (K_m_), turn over numbers (k_cat_), and the peak velocity (V_max_) of pure asparaginase. The V_max_ and K_m_ from Lineweaver-Burk plots were calculated by the aid of an equation obtained from a non-linear regression study of the curve. To get the k_cat_ value, the formula k_cat_ = V_max_ / [E] was utilized, where [E] represents the concentration of the enzyme used in the reaction. Each reaction was performed three times [31].

### Estimation of the antitumor activity and cytotoxicity of the recombinant L-asparaginase utilizing the viability assay compared to the marketed *E. coli* L-asparaginase

#### Cell lines and propagation

Human leukemia cancer cell line K-562, human hepatocellular carcinoma cell line HepG-2, and normal human lung fibroblast cell line MRC-5 were purchased from the American Type Culture Collection (ATCC, Rockville, MD). K-562 and HepG-2 cell lines were cultured in Rosewell Park Memorial Institute media (RPMI-1640) (Sigma Aldrich, USA) augmented with 10% inactivated fetal calf serum and 50 µg/ml gentamycin. The cells were sub-cultured twice a week and kept in a humidified environment with 5% CO_2_ at 37 °C [32–34].

MRC-5 cells were cultured in Dulbecco’s modified Eagle’s medium (DMEM), (Sigma Aldrich, USA), containing 50 µg/ml gentamycin, HEPES buffer (4-(2-hydroxyethyl)-1-piperazineethanesulfonic acid), 1% L-glutamine, and 10% heat-inactivated fetal bovine serum. The cells were sub-cultured 2 times a week and kept at 37 °C in a humidified environment with 5% CO_2_ [35].

#### Antitumor assay

The marketed L-asparaginase from *E. coli* (L-ASAP Neova, Biogene) and the recombinant L-asparaginase enzyme were assessed for their antitumor efficacy. The tumor cell lines, HepG-2 and K-562, were cultured for 24 h in 96-well plates of tissue culture (Corning^®^) with media containing 5 × 10^4^ cells/well. After that, the tested enzymes were added to three replicates of 96-well plates to produce 12 concentrations of each enzyme (six concentrations for each tumor cell), ranging from 0 to 10.352 IU/ml. For each plate of 96-well, six controls having medium or 0.5% DMSO were conducted in parallel. Using the MTT (3-[4,5-dimethylthiazol-2-yl]-2,5 diphenyl tetrazolium bromide) test, the number of viable cells was ascertained after incubation for 48 h [36]. To summarize, the media were taken out and fresh culture was added (RPMI 1640 medium that was free of phenol red) of 100 µl volume. Next, from 12 mM MTT stock solution, (5 mg MTT in 1 mL Phosphate Buffer Saline (PBS), 10 µl were introduced into each well, together with the untreated controls. Then the plates were kept at 37 °C for 4 h with 5% CO_2_. The extra media were removed carefully by blotting the plate gently, then DMSO was introduced into each well. The resultant solution was well mixed then shaken thoroughly utilizing the microtiter plate shaker to obtain a uniform solution. Finally, the plate was kept at 37 °C for 10 min. In order to ascertain the quantity of live cells, the optical density (OD) was then measured at 590 nm using a microplate reader (SunRise, TECAN, Inc., USA).

Based on the formula [(ODt/ODc)]x100, the percentage of viability was determined where, ODt represents the mean optical density of the treated wells with the tested L-asparaginase, and ODc represents the mean optical density of the cells that have no treatment [31]. For each treated tumor cell line, a survival curve is obtained by plotting the concentration of the enzyme and the remaining cells. The concentration needed to produce harmful influence in 50% of intact cells is known as the 50% inhibitory concentration (IC50) was calculated the dose response curve graphic plots using Graphpad Prism software (San Diego, CA. USA) [37, 38].

#### Cytotoxicity assay

To assess the safety of the recombinant L-asparaginase, the cytotoxicity of the recombinant *S. maltophilia* L-asparaginase compared to the marketed L-asparaginase from *E. coli* was investigated on normal human lung fibroblast cells. In 100 µl of the growth media, MRC-5 cells were seeded at 1 × 10^4^ concentration per well in a plate of 96-well. For a duration of 24 h, the microtiter plates were kept at 37 °C in an incubator with 5% CO_2_. Confluent cell monolayers were pipetted into microtiter plates with flat-bottomed 96-well (Falcon, NJ, USA), and two-fold serial dilutions of the tested L-asparaginases were added. For each test enzyme concentration, 3 wells were used. Control cells were cultured in the presence or absence of DMSO and without the test enzyme. As previously indicated, a colorimetric approach using MTT assay was used to measure the yield of the viable cell following the cells incubation [37, 39].

#### Evaluation of the immunogenicity for recombinant L-asparaginase and the marketed L-asparaginase from *E. coli*

The immunogenicity of L-asparaginase of *S. maltophilia* and the FDA approved L-asparaginases from *Erwinia chrysanthemi* and *E.coli* previously in silico predicted using the Maximum Likelihood technique of the JTT matrix-based model [11]. Immunogenicity was validated using an in vivo assay. Male BALB/c mice that were 6–8 weeks old and its weight ranged from 20 to 30 gm were used in this experiment. Animals were kept in open cages in a climate controlled by air conditioner, with a temperature of 25 °C and 12 h light and dark cycles. They were fed with water and antibiotic-free food containing 20% protein, 6.5% ash, 3.5% fat, 5% fiber, and a blend of vitamins. The study was approved by the Research Ethics Committee of the Faculty of Pharmacy Ain Shams University, Egypt (ENREC – ASU 2020 − 101).

The mice groups (*n* = 12/group) were intraperitoneally injected with 250 U/kg of either marketed L-asparaginase or recombinant *S. maltophilia* L-asparaginase 2 times per week for four weeks [40]. The 3rd group (n = 12/group)was designated as the control group and given 50 mM Tris HCl buffer pH 8.6 [41]. The developed antibodies in sera were measured using the ELISA method utilizing anti-mouse anti-IgG Horse Radish Peroxidase conjugate. The assay was performed at room temperature and 0.05% (PBS)-Tween 20 plus 5% Skimmed milk was used as blocking buffer [42].

The assay parameters and procedures were as follows: The L-asparaginase-coated plates were blocked for 2 h, followed by a thorough washing with PBS-Tween 20, then kept with the diluted serum samples for 2 h, followed by another washing step and the plates were kept with anti-mouse anti-IgG Horse Radish Peroxidase conjugate for 1 h then washed once more. The plates with the substrate (3, 3′, 5, 5′-tetramethylbenzidine) were incubated for the development of color. The stop solution was added, and the plate reader was utilized to determine the absorbance at 450 nm within 30 min. The absorbance of blank sample was subtracted from the sample’s absorbance value to determine the value that corresponded to the IgG antibody level. The IgG titre was determined by taking the highest dilution’s reciprocal whose reading was more than twice the control value [43].

#### Analyses of statistical data and graphical displays

Three sets of experiments were carried out, and the standard deviation and mean values (shown by error bars) were recorded. GraphPad Prism 8 software (GraphPad Inc., La Jolla, CA, USA) was used to do the Student t-test, one-way ANOVA, Tukey’s Multiple Comparison Tests, and two-way ANOVA.

## Results

### Cloning and heterologous expression of *S. maltophilia* L-asparaginase

**Fig. 1:**
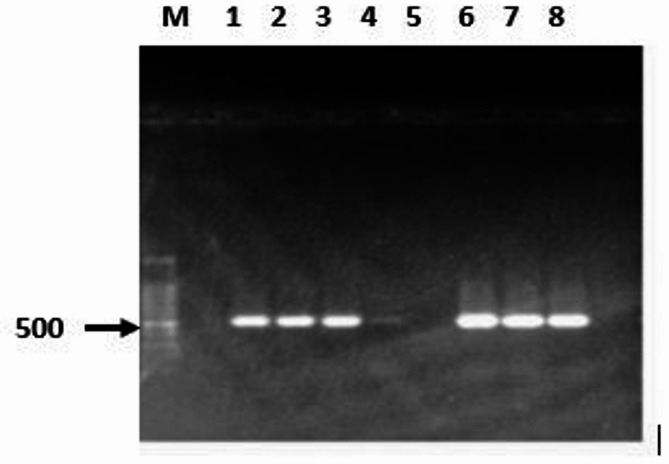
Colony PCR gel electrophoresis for detection of pET 22b(+) T7-L-asparaginase. The first lane (M) 1Kb ladder, Lanes (1,2,3,6,7,8) L-asparaginase positive clones (plasmid containing the gene of interest), Lanes 4 & 5 negative clones (plasmid without the gene of interest)

Genomic DNA from *S. maltophilia* isolate was used to extract the gene coding for L-asparaginase enzyme. Thus, the 500 bp PCR amplicon size carrying *Nde*I */Bam*HI restriction sites at the 5’ and 3’ regions, respectively was cloned using the pET 22b (+) T7 vector resulting in intermediate plasmid pET 22b (+) T7-asparaginase. The constructed recombinant plasmid was used for the transformation into *E. coli* DH5α. Then, screening of the positive clones was performed by colony PCR using gene specific primers. A number of colonies were found to have the L-asparaginase gene and yielded positive amplifications with size 500 bp (Fig. 1).

### Studying the influence of various factors on the expression and productivity of recombinant L-asparaginase produced by *E. coli* BL21 (DE3)-pET 22b(+)-ASP

#### Effect of incubation temperature

**Fig. 2:**
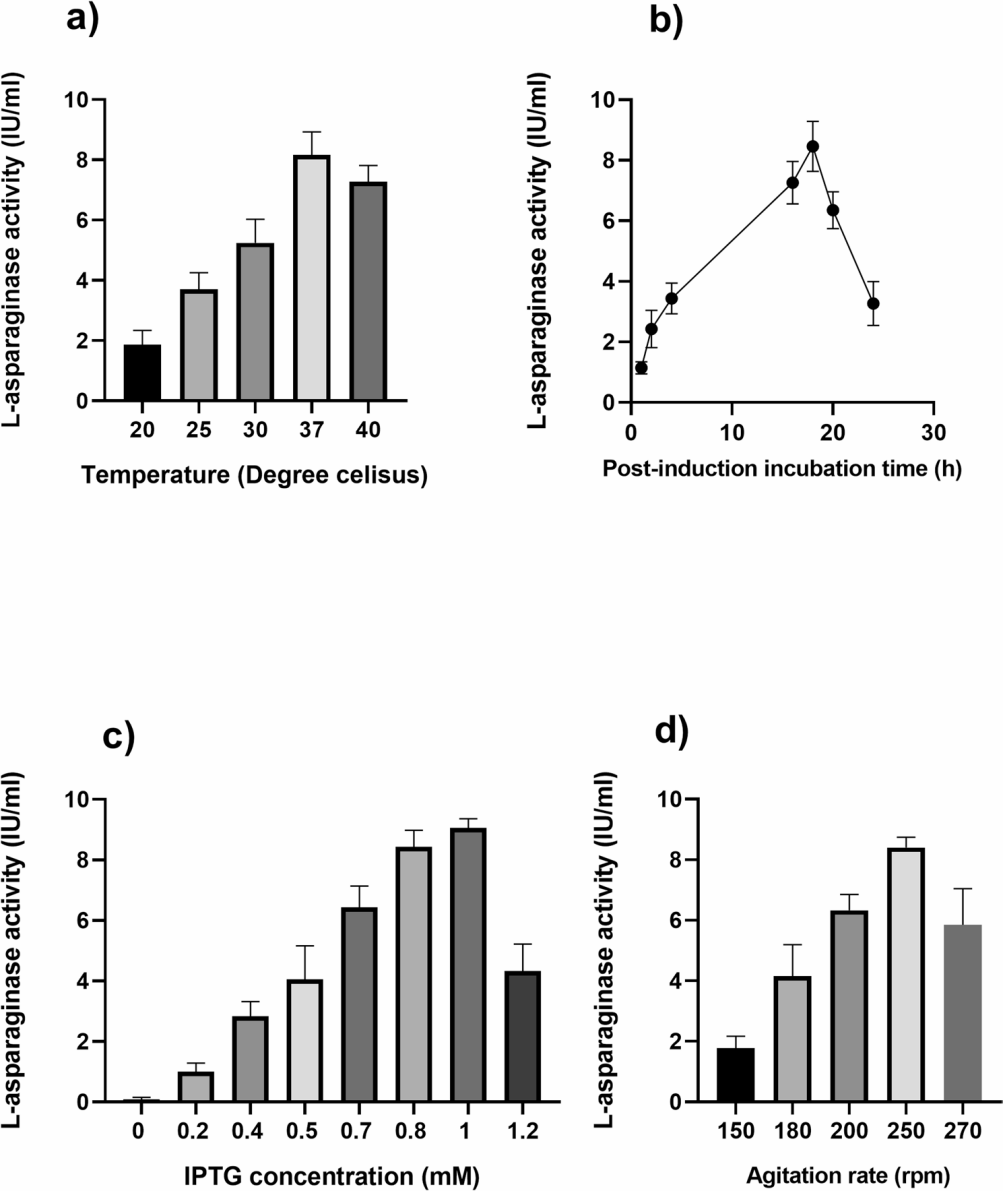
Influence of various factors on L-asparaginase expression by *E. coli* BL21 (DE3). Incubation temperature (**a**), post-induction incubation time (**b**), IPTG concentration (**c**), and agitation rate (**d**). The expression of L-asparaginase was expressed in the terms of catalytic activity

Expression of recombinant L-asparaginase was evaluated at different incubation temperatures ranging from 20 to 40 °C with 1 mM IPTG concentration overnight incubation. As shown in Fig. 2a, L-asparaginase expressed at 20 °C and above, reached the maximum value at 37 °C and decreased slightly at 40 °C.

#### Effect of incubation time after IPTG addition

The influence of incubation time following IPTG addition on the productivity of recombinant L-asparaginase was studied by measuring the expression at different time intervals at 37 °C with 1 mM IPTG concentration. The results revealed that expression of L-asparaginase gradually increased by increasing incubation time till reaching its maximum induction after 18 h followed by a steady decrease in the expression (Fig. 2b).

#### Effect of different IPTG concentrations

Different concentrations of IPTG were tested to evaluate their effects on the overnight expression of recombinant L-asparaginase at 37 °C. The results revealed that 1 mM concentration of IPTG produced the highest L-asparaginase expression. By increasing IPTG concentration there was a decrease in the expression. (Fig. 2c).

#### Effect of agitation rate

Recombinant L-asparaginase expression was studied at different agitation rates. The results showed maximum L-asparaginase expression at 250 rpm and low expression levels at agitation speeds below and above this value (Fig. 2d).

#### Optimization of recombinant L-asparaginase expression produced by *E. coli* BL21 (DE3)-pET22b(+)-ASP using RSM

The effect of the four studied variables (incubation temperature, induction incubation time, IPTG concentration, and agitation rate) and their possible interactions were investigated. Corresponding L-asparaginase productivity (IU/ml) was determined in each run. The experiments deduced from the model were 27 experiments including 3 replicates at the center points of variables for optimization of L-asparaginase productivity. The response surface model results along with predicted, observed, residual values are shown in Table 3. The anticipated values could be derived as follows based on the regression equation of the mathematical model: L-asparaginase productivity = 5.62557–2.28751*A + 1.75955*B − 1.4003*C + 0.0804516*D + 0.0398456*A^2^ − 0.010055*A*B + 0.101667*A*C + 0.00226667*A*D − 0.0310975*B^2^ + 0.1675*B*C − 0.0018*B*D − 2.77637*C^2^ − 0.00234286*C*D − 0.000241812*D^2^.

Where A was the incubation temperature, B was the incubation time, C was IPTG concentration and D was agitation rate.

**Fig. 3:**
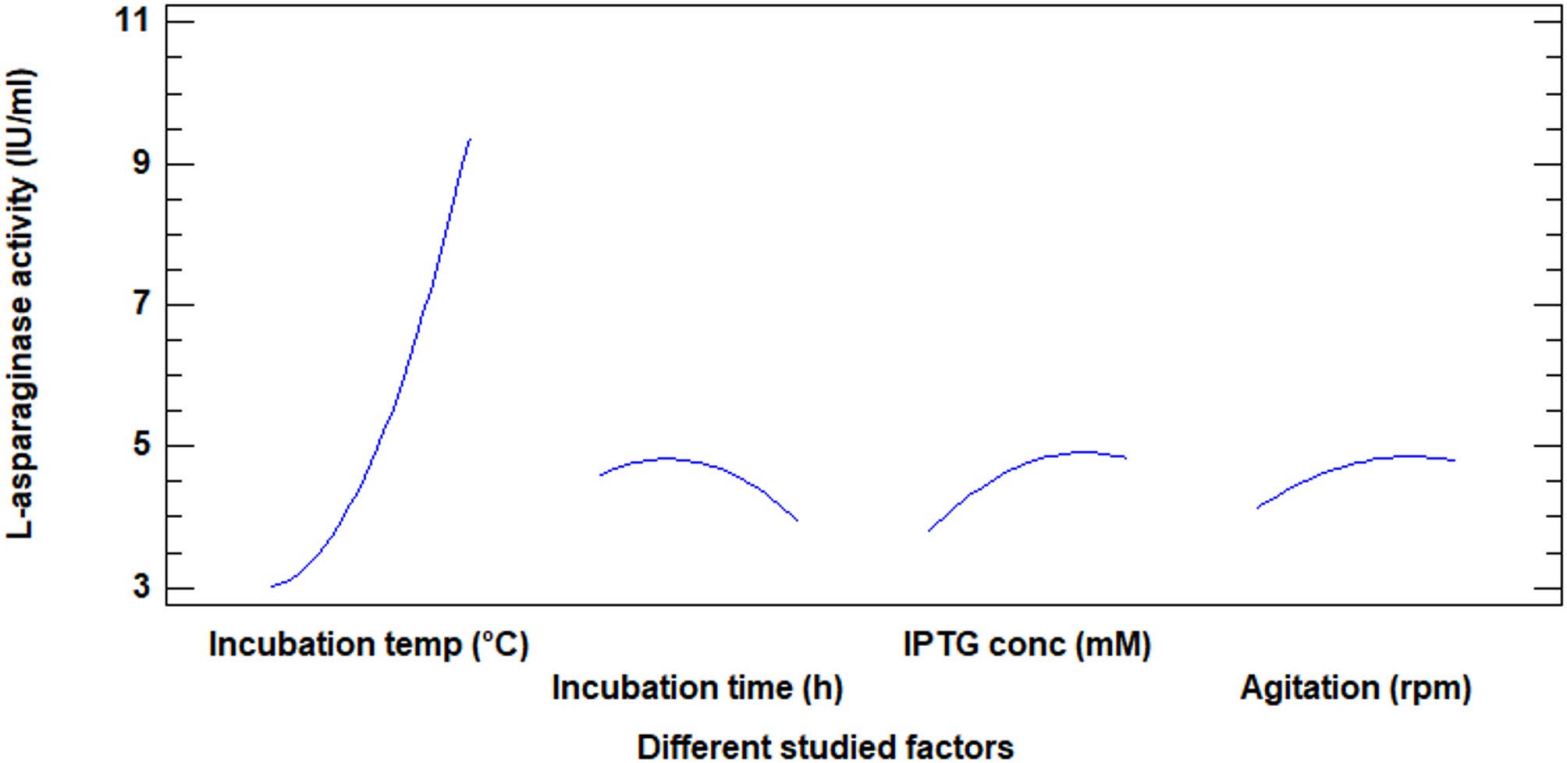
Main effects plot for the influences of the tested factors on the activity of recombinant L-asparaginase in *E. coli* BL21 (DE3)

Table 4 displays the *p*-values and estimated factor effects for the ANOVA-generated findings. While The process variables that were determined to be significant for the expression of L-asparaginase are listed in Table 5. A model’s fit was assessed using the model’s regression coefficient(R2). The R^2^ was computed to be 0.9815, remarking that the model could describe 98.15% of the variability. There is just 1.85% of the overall variation that the model couldn’t account for. The adjusted R-squared value is 96%, making it better suited for comparing models with varying numbers of independent variables. Based on the estimate’s standard error, the residuals’ standard deviation of 0.48. The residuals have an average value of 0.23 mean absolute error (MAE). The residuals are tested using the Durbin-Watson (DW) statistic to see if any significant correlation exists. Serial autocorrelation is not present in the residuals at the 5.0% significance level since the *p*-value is higher than 5%. Figure 3 demonstrates the major effects plot of the variables on the studied responses. If there is a deviation from zero and the *p*-value is less than 0.05, the factor influence is considered significant.

**Table 3:** Observed, predicted, and residual values obtained from RSM experimental runs for optimization of process variables of Recombinant L-asparaginase production by the Recombinant *E. coli* BL21 (DE3)-pET-22b(+)-ASP

Runs	Temperature (°C)	Time (h)	IPTG concentration (mM)	Agitation (rpm)	L-asparaginase productivity IU/ml		
					Observed value	Predicted value	Residual value
1	25	20	0.2	215	2.368	2.31493	0.05307
2	37	20	0.2	215	8.224	8.18871	0.03529
3	31	16	1	215	4.688	4.39485	0.29315
4	37	20	0.6	250	9.36	9.88978	-0.52978
5	31	16	0.2	215	3.952	3.91805	0.03395
6	31	20	0.2	180	3.456	3.16169	0.29431
7	25	20	0.6	180	2.976	2.87466	0.10134
8	31	24	1	215	3.808	4.27039	-0.46239
9	31	24	0.2	215	2	2.72159	-0.72159
10	37	20	0.6	180	7.84	8.28445	-0.44445
11	31	24	0.6	250	3.808	3.71866	0.08934
12	37	24	0.6	215	9.136	8.31423	0.82177
13	25	20	1	215	2.848	2.83973	0.00827
14	25	20	0.6	250	2.592	2.57599	0.01601
15	31	20	0.6	215	5.248	4.768	0.48
16	31	20	0.2	250	4.1856	3.88063	0.30497
17	31	20	0.6	215	4.56	4.768	-0.208
18	25	24	0.6	215	2.69328	2.43508	0.2582
19	31	20	1	250	4.9184	4.82783	0.09057
20	31	16	0.6	180	3.68	3.72578	-0.04578
21	37	20	1	215	9.68	9.68951	-0.00951
22	31	20	0.6	215	4.496	4.768	-0.272
23	31	16	0.6	250	4.912	4.88311	0.02889
24	37	16	0.6	215	9.584	9.45732	0.12668
25	31	24	0.6	180	3.584	3.56933	0.01467
26	31	20	1	180	4.32	4.24009	0.07991
27	25	16	0.6	215	2.176	2.61289	-0.43689

**Table 4:** Estimated effects of factors affecting Recombinant L-asparaginase production in *E. coli* BL21 (DE3) associated with *p*-values for response

Effect	Estimate	*p*-Value
A: Incubation temp	6.36179	**0.000**
B: Incubation time	-0.660453	**0.036**
C: IPTG conc	1.0128	**0.0035**
D: Agitation	0.653333	**0.0377**
AA	2.86888	**0.000**
AB	-0.48264	0.3388
AC	0.488	0.3337
AD	0.952	0.073
BB	-0.99512	**0.0353**
BC	0.536	0.2902
BD	-0.504	0.3187
CC	-0.88844	0.0558
CD	-0.0656	0.8945
DD	-0.59244	0.1833

**Table 5:** Process variables having significant influence on the productivity of Recombinant L-asparaginase in *E. coli* BL21 (DE3) in descending order

Order	Process variable
1	Incubation temperature
2	Square term of Incubation temperature
3	IPTG concentration
4	Square term of Incubation time
5	Incubation time
6	Agitation rate

**Fig. 4:**
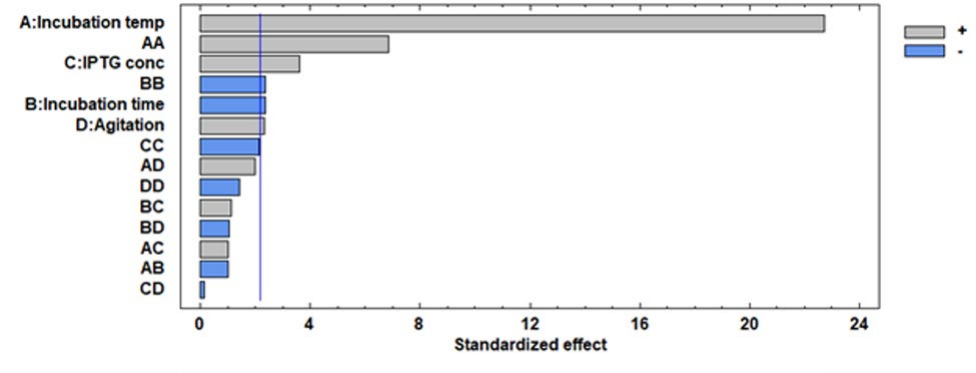
Standardized Pareto chart displaying the effect of the tested factors on L-asparaginase productivity by recombinant *E. coli* BL21 (DE3). In the current model, bars indicate significant interactions ranging from the highest to the lowest, irrespective of the impact sign. The vertical line denotes the reference line, and any factor beyond it has a significant effect at the significance level of α = 0.05. The standardized effect’s positive or negative sign indicates whether it had a positive or negative impact on the replies

**Fig. 5:**
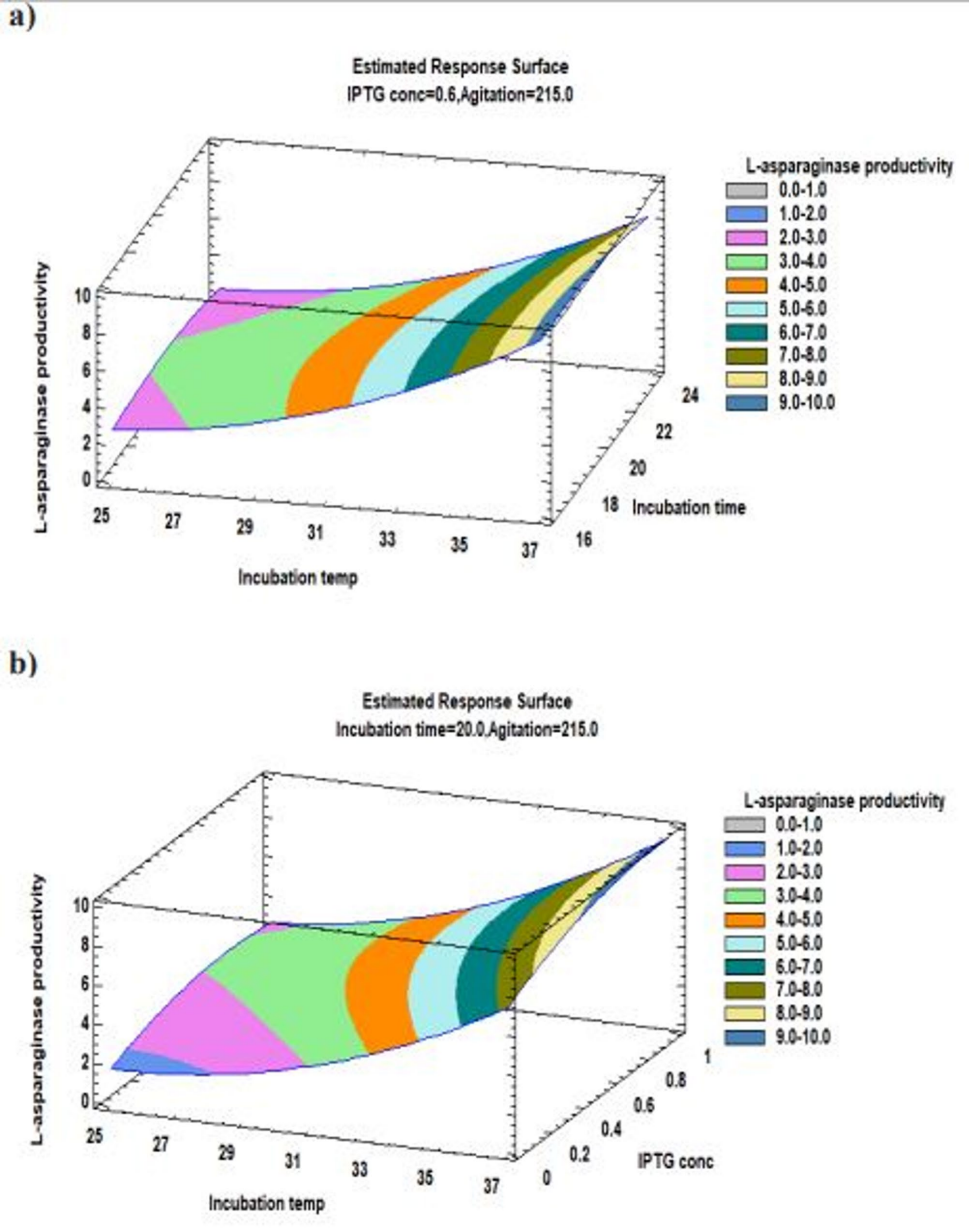
Response surface plot of the process variables’ optimization for the production of recombinant L-asparaginase in *E. coli* BL21 (DE3) showing the influence of **a** the interactions between incubation temperature (°C) and post-induction incubation time (h); **b** the interactions between incubation temperature (°C) and IPTG concentration (mM) on L-asparaginase productivity

**Fig. 6:**
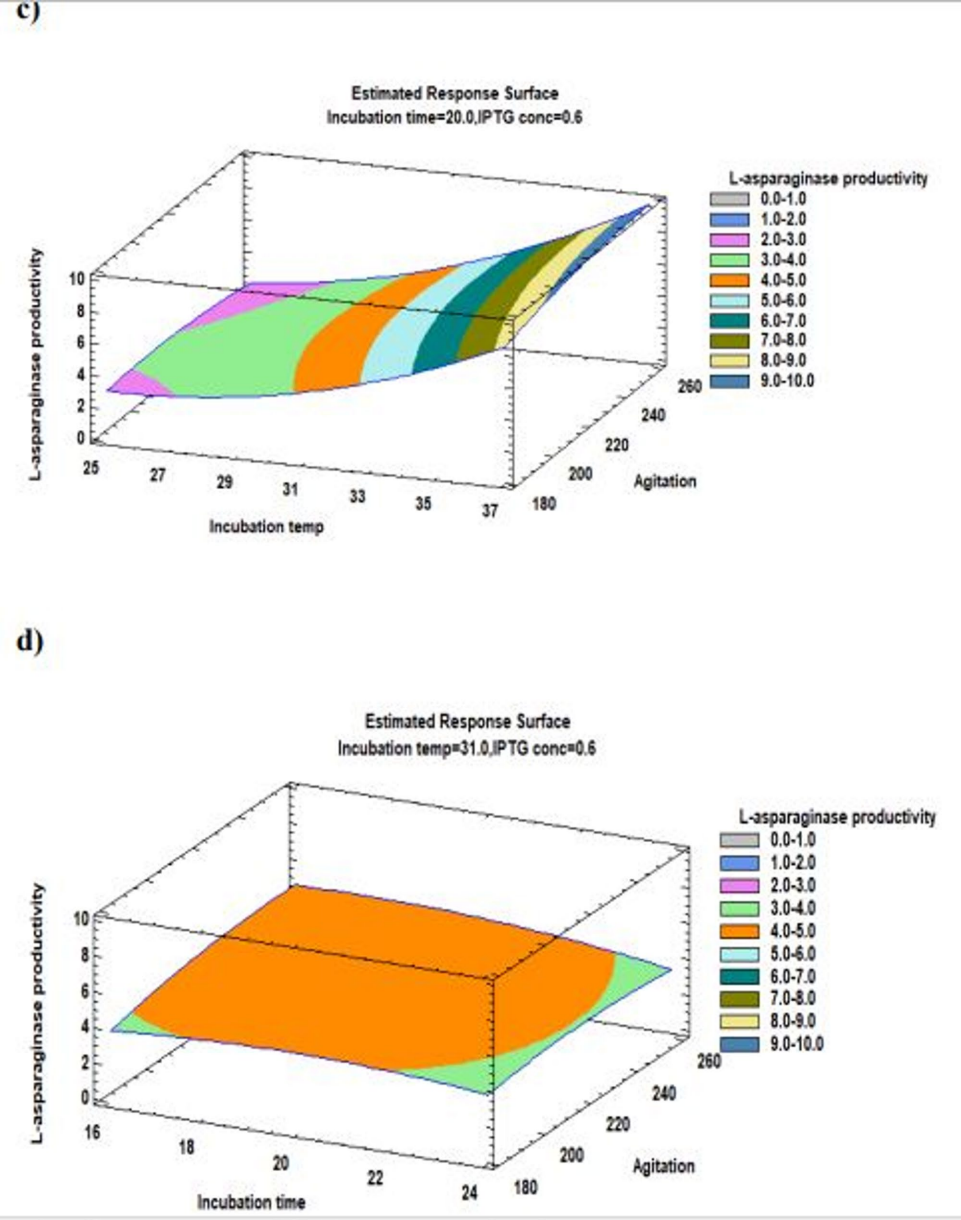
Response surface plot of the process variables’ optimization for the production of recombinant L-asparaginase in *E. coli* BL21 (DE3) showing the influence of **c** the interactions between incubation temperature (°C) and agitation rate (rpm) on L-asparaginase productivity; **d** the interactions between agitation rate (rpm) and post-induction incubation time (h) on L-asparaginase productivity

**Fig. 7:**
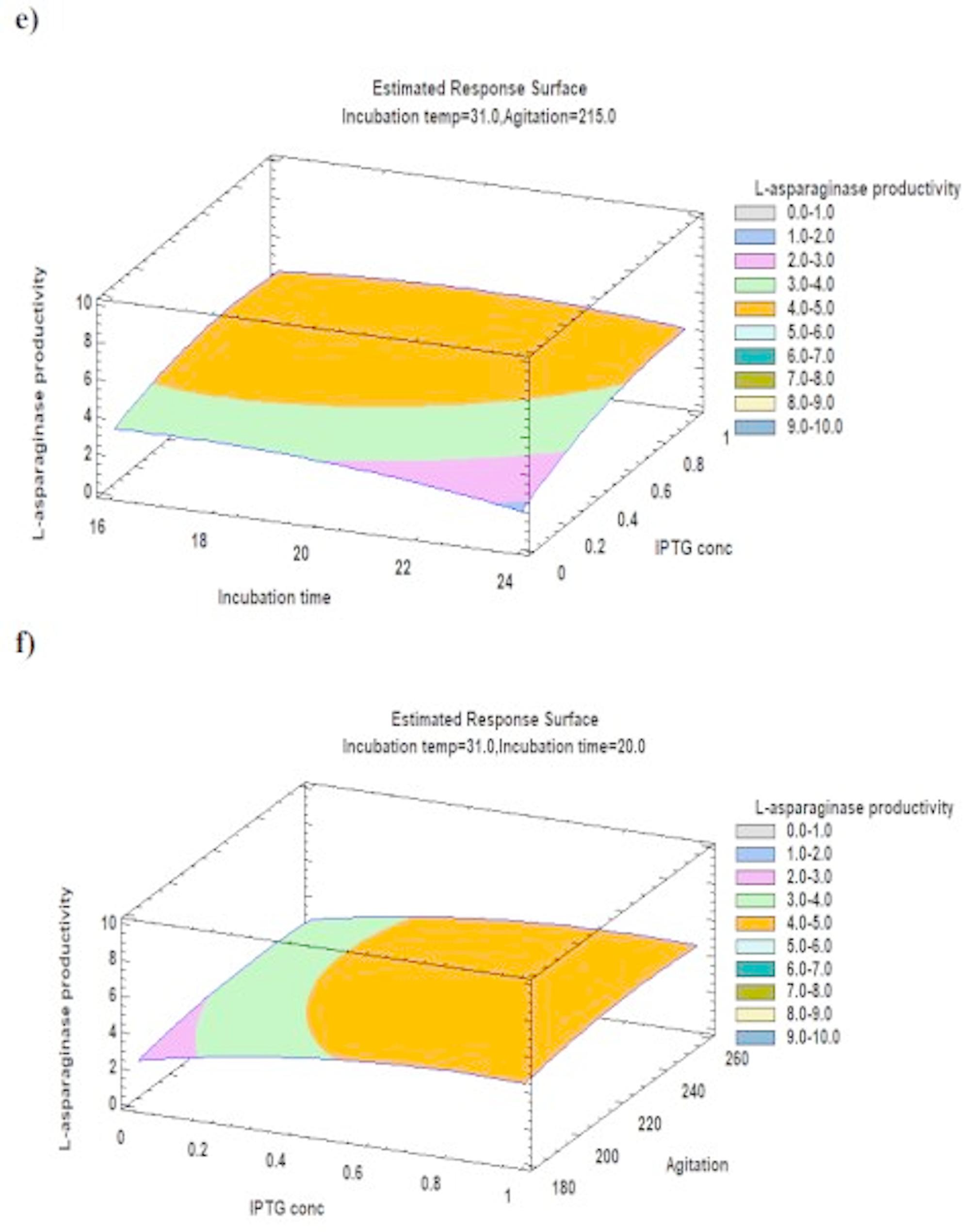
Response surface plot of the process variables’ optimization for the production of recombinant L-asparaginase in *E. coli* BL21 (DE3) showing the influence of; **e** the interactions between IPTG concentration (mM) and post-induction incubation time (h); **f** the interactions between IPTG concentration (mM) and agitation rate (rpm) on L-asparaginase productivity

The Pareto chart illustrates the relationship between various variables and the L-asparaginase productivity along with the major ones represented in Fig. 4. Furthermore, a three-dimensional response surface plots illustrate the effect of each component on the productivity of L-asparaginase at each of the factor levels under investigation. The 3D response surface plots are the graphical representations of the regression equation (Figs. 5, 6 and 7). Response surface plots [RSPs] represented in Fig. 5a and b exhibit the influence of temperature and its interactions with time and IPTG concentration, respectively. The interactions revealed the predicted L-asparaginase expression at its maximum level of 9.53381 IU/ml at temperature 37 °C for 17 h, 9.7001 IU/ml at the same temperature (37 °C) and 0.944 mM IPTG. On the other hand, RSPs displayed in Fig. 6c and d demonstrate the effect of interaction of agitation rate with incubation temperature and its interaction with induction incubation time, respectively. The results revealed the predicted L-asparaginase expression of 9.887 IU/ml could be achieved at 250 rpm and 37 °C and 4.946 IU/ml at the same agitation rate for 16.8 h, respectively. While RSPs presented in Fig. 6e and f present the effect of interactions of IPTG concentration with induction incubation time and its interaction with agitation rate, respectively. The interactions show the predicted L-asparaginase expression at its maximum level of 4.71867 IU/ml utilizing 0.856 mM IPTG and 16.8 h and L-asparaginase expression of 4.9029 IU/ml at concentration of 1 mM and 231 rpm, respectively.

The fundamental goal of using RSM is to specify the best value for each variable in order to increase the researched response to its maximum level. Based on the employed model, the maximum predicted value of L-asparaginase productivity is 10.4 IU/ml, which can be achieved at 37 °C, 250 rpm, and 0.83 mM IPTG concentration for 17 h.

#### Validation of optimized conditions resulted from RSM for the expression of the recombinant L-asparaginase by *E. coli* BL21 (DE3)-pET22b(+)-ASP

L-asparaginase productivity of the recombinant enzyme was assessed utilizing the optimized conditions (37 °C, 250 rpm, 0.83 mM IPTG concentration, and 17 h incubation time) that were obtained from the design used to validate the optimization of the RSM. The L-asparaginase productivity was found to be 10.3 IU/ml which is very close to the predicted L-asparaginase production value of 10.4 IU/ml.

#### Purification of the recombinant L-asparaginase protein

**Fig. 8:**
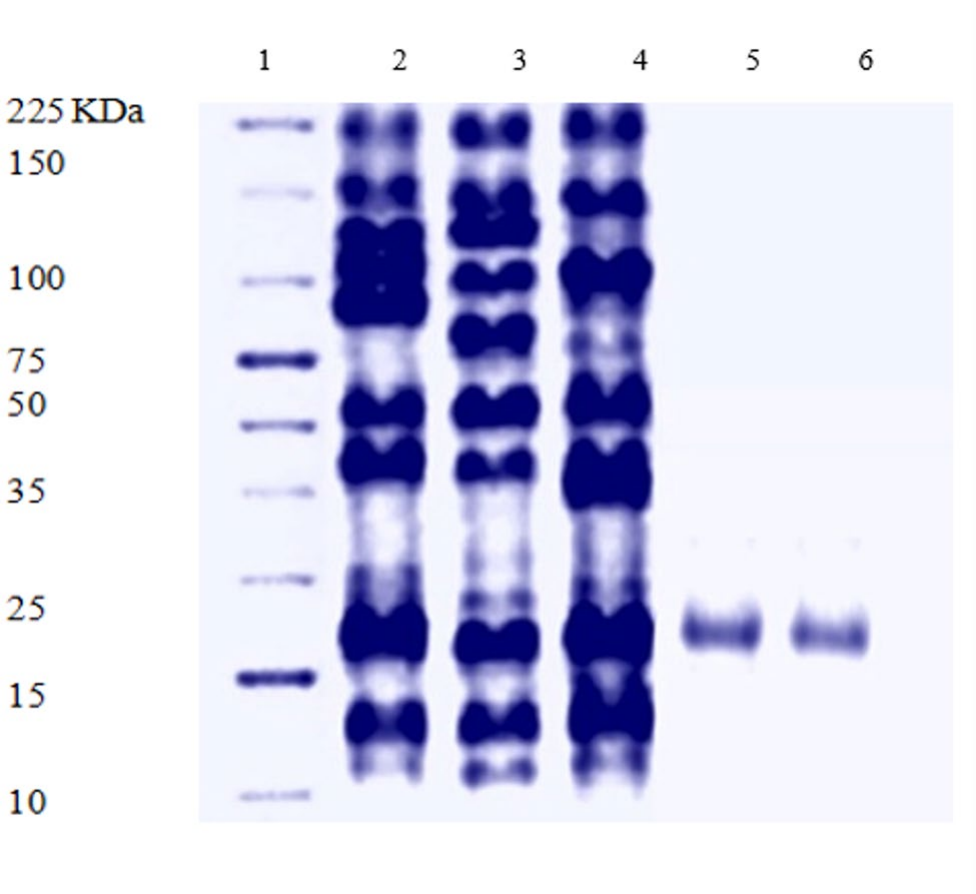
SDS-PAGE of the purified recombinant L-asparaginase produced in *E. coli* BL21 (DE3)-pET22b(+)-ASP. Lane 1: Protein marker; Lane 2: recombinant L-asparaginase cell lysate; Lane 3: crude extract Lane 4: the wash through of Ni-NTA column; Lane 5 & 6: purified recombinant L-asparaginase

The recombinant plasmid (pET22b(+)-ASP) was transformed into *E. coli* BL21 (DE3). The resulting recombinant protein from *E. coli* BL21 (DE3)-pET22b(+)-ASP culture was purified by the aid of Ni-NTA columns. SDS-PAGE was conducted to determine the molecular weight of the protein where a definite band with expected size 17 KDa was visualized (Fig. 8). Bradford assay was used to determine the protein concentration of the cell lysate and the purified enzyme. It showed that the protein concentration of the cell lysate was 6399.2 µg/ml while that of the purified enzyme was 4024 µg/ml.

#### Assessment of the kinetic parameters of the recombinant L-asparaginase produced by *E. coli* BL21 (DE3)-pET22b(+)-ASP

**Fig. 9:**
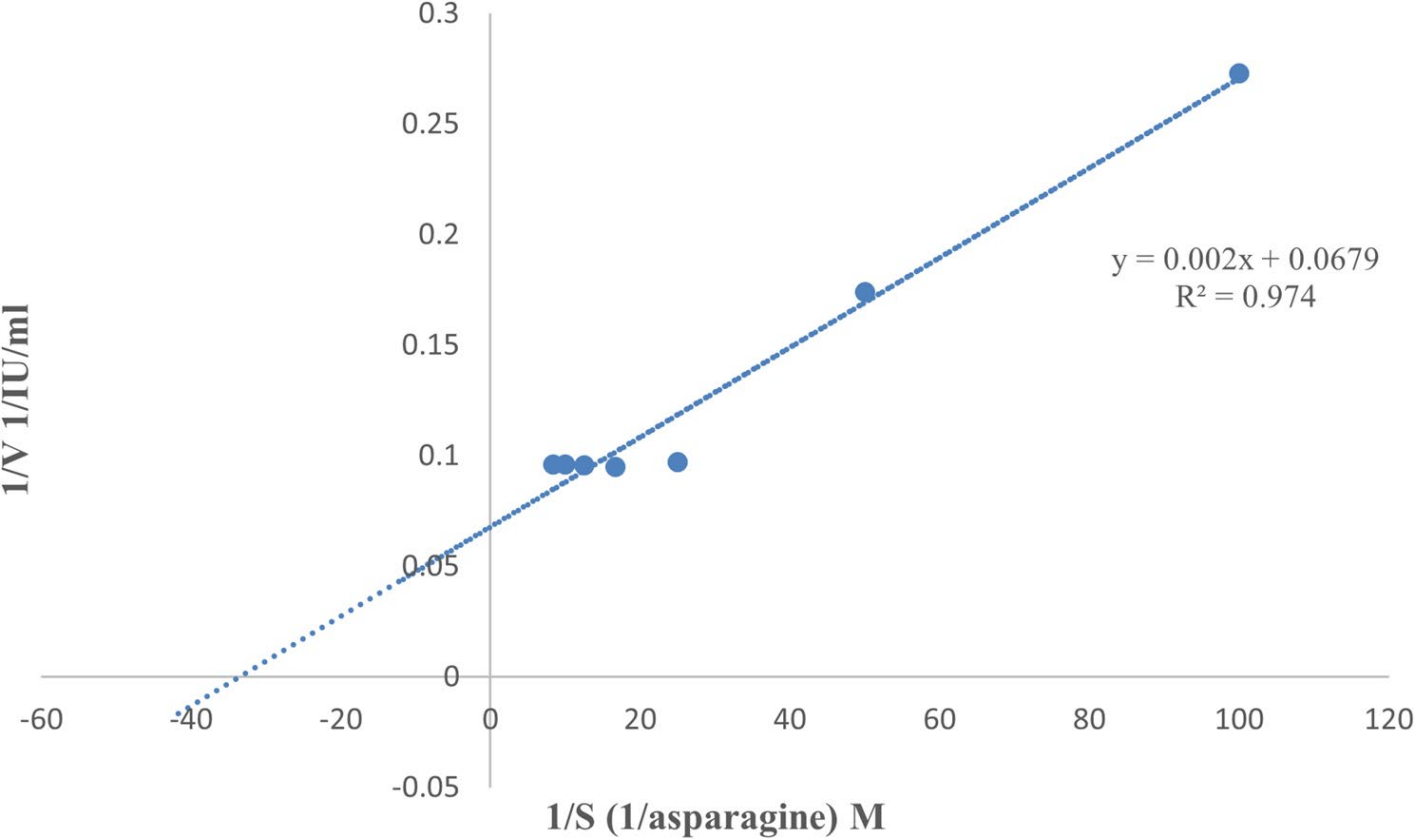
The Lineweaver Burk plot of the recombinant L-asparaginase produced by *E. coli* BL21 (DE3)-pET22b(+)-ASP for estimating enzyme kinetic parameters

As illustrated in Fig. 9, the kinetic parameters of the recombinant L-asparaginase were ascertained by creating a Lineweaver-Burk plot using enzyme activity values calculated at the optimum temperature and pH. The K_m_ was 2.94 × 10^− 2^ M, V_max_ 14.73 IU/ml while the k_cat_ was 1.397 S^− 1^.

#### Antitumor activity and cytotoxicity of the purified recombinant L-asparaginase produced by *E. coli* BL21 (DE3)-pET22b(+)-ASP in comparison to the marketed *E. coli* L-asparaginase

**Fig. 10:**
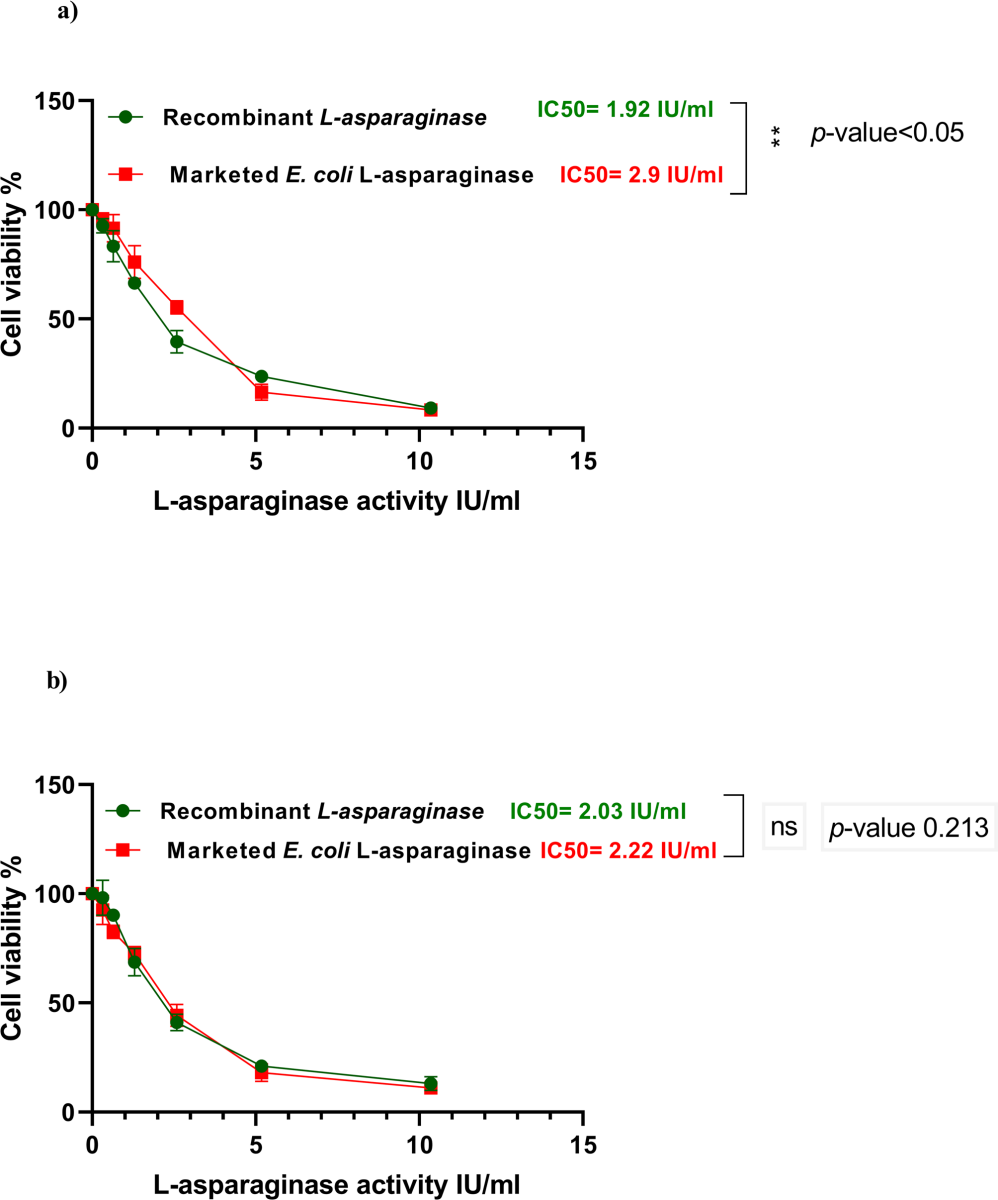
Antitumor activities of recombinant L-asparaginase produced by *E. coli* BL21 (DE3)-pET22b(+)-ASP and the marketed *E. coli* L-asparaginase. **a** Antitumor activity on HepG-2 cells and **b** Antitumor activity on K-562 cells. The results denote the average of the data received from three experiments and the error bars show standard deviation. The results were statistically analyzed using student t-test

**Fig. 11:**
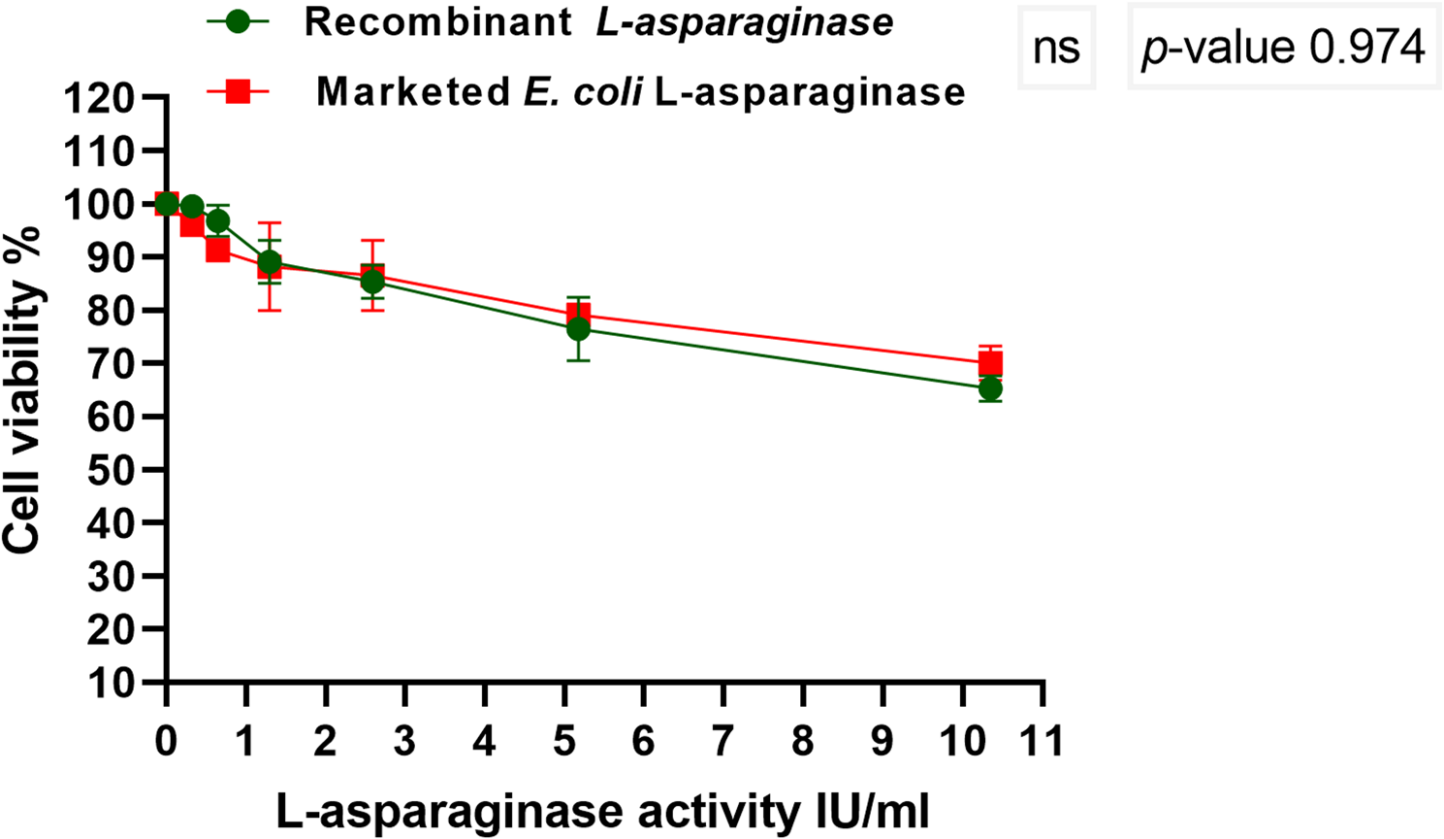
Cytotoxic activity of recombinant L-asparaginase produced by *E. coli* BL21 (DE3)-pET22b(+)-ASP and the marketed *E. coli* L-asparaginase on MRC-5 cells. The results denote the average of the data received from three experiments and the error bars show standard deviation

The MTT assay was utilized to evaluate the antitumor activity of both the marketed *E. coli* L-asparaginase and the purified recombinant L-asparaginase. The recombinant L-asparaginase was found to be efficient against K-562 and HepG-2 tumor cells at low concentrations; at 2.58 IU/ml, 61% of HepG-2 cells and 59% of K-562 cells were destroyed by the enzyme. The results indicate dose-dependent cytotoxicity. The recombinant L-asparaginase had a lower IC50 for HepG-2 than that of the marketed one; the IC50 values for the recombinant and marketed L-asparaginase were found to be 1.92 IU/ml and 2.9 IU/ml, respectively. There was significant difference between the two enzymes at *p*-value < 0.05. In contrast, the recombinant enzyme’s IC50s for K-562 cells has no significant difference from that of the marketed enzyme, with the former’s being 2.03 IU/ml and the latter’s being 2.22 IU/ml (Fig. 10a and b). Furthermore, it was found that recombinant L-asparaginase has no cytotoxic effect on normal cell MRC-5, with non-significant difference between its IC50 and that of the marketed *E. coli*, as illustrated in Fig. 11.

#### Immunogenicity of the recombinant L-asparaginase produced in *E. coli* BL21 (DE3)-pET22b(+)-ASP as compared to the marketed L-asparaginase from *E. coli*

**Fig. 12:**
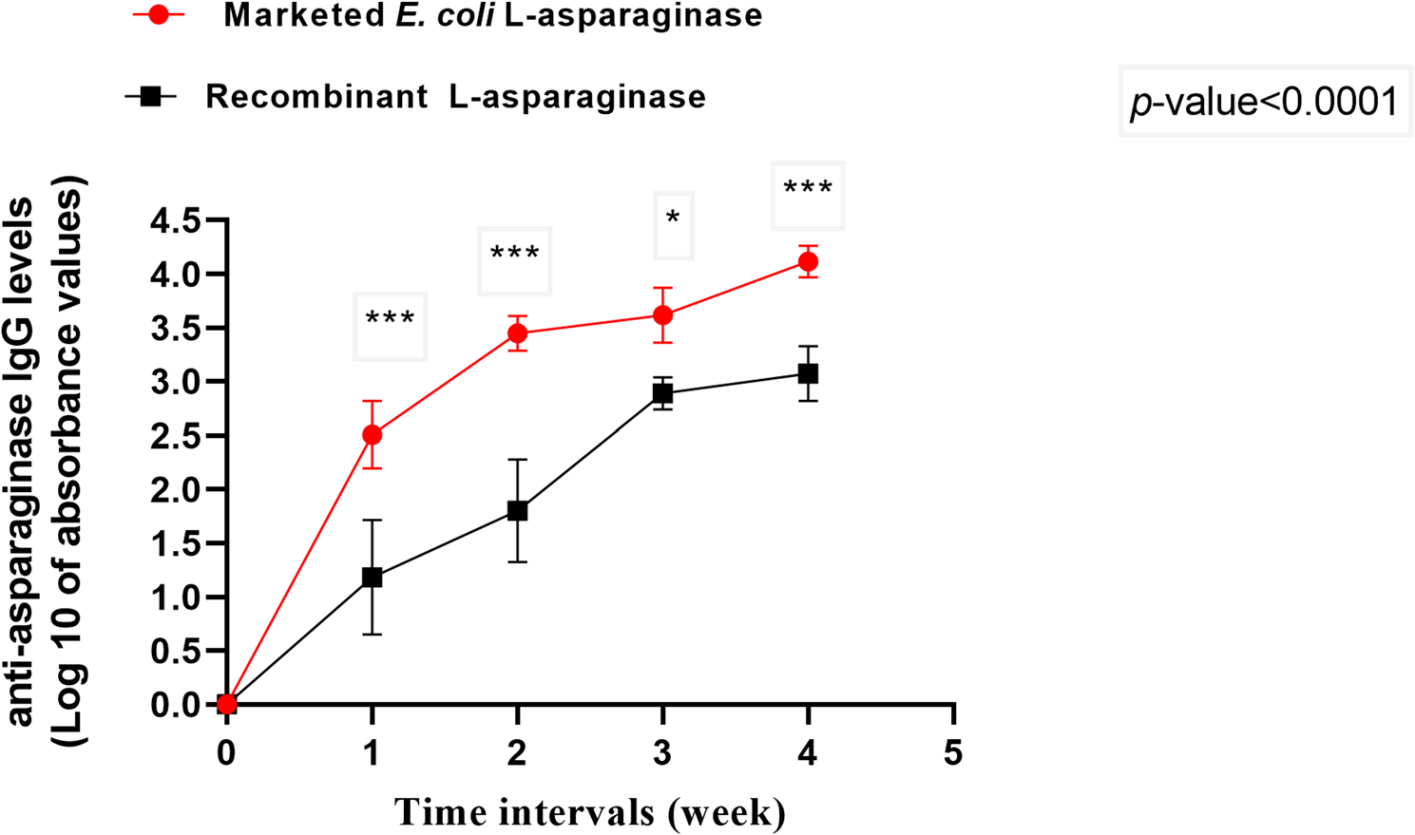
The evolution of anti-L-asparaginase IgG over time in mice treated with marketed L-asparaginase and the recombinant L-asparaginase produced by *E. coli* BL21 (DE3)-pET22b(+)-ASP presented as log 10 of absorbance values as determined by ELISA technique. The results denote the average of the data received from three independent experiments and the error bars show standard deviation. The results were statistically analyzed by the aid of the Two-way ANOVA Tukey’s Multiple Comparison Test. The results exhibit significant difference between the anti L-asparaginase IgG levels of the two tested enzymes along the different time intervals in the immunogenicity of the investigated L-asparaginases compared to the marketed one (*p*-value < 0.0001)

ELISA technique was utilized to assess the levels of IgG against L-asparaginase in sera of challenged mice and to characterize the immunogenicity profiles of the recombinant and the marketed *E. coli* L-asparaginases in mice. The enzymes were injected intraperitoneally in several mice groups, the anti-L-asparaginase IgG levels were assessed at various intervals. Figure 12 illustrates the results, which demonstrate gradual rise in the anti-L-asparaginase IgG titre for each provided L-asparaginase. The outcomes showed a significant difference (*p*-value < 0.0001) in the IgG titre levels between the marketed L-asparaginase and the recombinant L-asparaginase, indicating that the latter is less immunogenic. These outcomes confirmed our earlier bioinformatics results showing that the L-asparaginase extracted from *S. maltophilia* was less immunogenic than that from *E. coli* reference one [11].

## Discussion

ALL is the most prevalent malignancy in children especially in ages 2–4 [44]. L-asparaginase is a tetrameric enzyme belongs to the amidohydrolases family and responsible for breaking down L-asparagine into ammonia and L-aspartic acid. It had been one of the major chemotherapeutic medicines for the treatment of ALL since it was discovered to be an anticancer medication [45]. It has been approved for clinical usage for the treatment of ALL in children [46]. Currently, L-asparaginase preparations were derived from *E.coli* and *Erwinia chrysanthemi*.However, they had immunogenicity problems and low yield production [45].

According to our formerly mentioned bioinformatics studies, L- asparaginase extracted from *S. maltophilia* has reduced immunogenicity in comparison to the ones from *E.coli* and *Erwinia chrysanthemi* origin [11]. This study aimed at cloning and production of L- asparaginase extracted from *S. maltophilia* in *E. coli* as a host organism. The recombinant and marketed L-asparaginases were assessed for their antitumor activity as well as their immunogenicity using animal model, besides their catalytic parameters.

Recombinant protein technology has enabled recombinant protein-based biopharmaceuticals expression employing bacterial, mammalian, yeast, transgenic plant, insect, and transgenic animal cells [47–50]. *E. coli* is broadly used for a variety of gene-cloning experiments as well as molecular DNA cloning as the entire genome of *E. coli* has been completely sequenced, and the methods for manipulating it are developing quickly [51]. A variety of mutations have been identified in *E. Coli* DH5α, including the Lacz delta m15 mutation that enables blue-white screening for recombinant cells, the enda1 mutation that lowers endonuclease degradation to guarantee higher plasmid transfer rates, and the reca1 mutation that lessens homologous recombination for a more stable insert.

The pET 22b (+) vector containing T7 promoter, the His-tag, and multiple cloning site (MCS) was used for cloning of L-asparaginase gene. This system has the benefit of having a relatively high product yield a few hours after induction begins. This is related to the selectivity and high activity of the T7 RNA polymerase, which directs all of the cell’s resources to express the target gene [52]. The His-tag fragment additionally permits quick and simple purification utilizing nickel columns.

In this study, L-asparaginase gene was successfully cloned into the multi-cloning site between the restriction sites; *Bam*H1 (5′GGATCC3′) and *Nde*1 (5’CATATG3’) and upstream of the T7 promoter via PCR. The recombinant plasmid (pET 22b (+) - L-asparaginase) was then transformed into *E.coli* DH5α for the propagation process as according to different researches DH5α effectively raises the plasmid count while maintaining the DNA product’s stability [18, 20, 22, 53, 54].

Colony PCR is a technique that uses a single colony of organisms to amplify DNA fragments without isolating pure form of DNA and bypass different steps including DNA isolation or restriction digestion [16, 17, 55–57]. This method is used to ensure the correct assembly of cloned DNA in plasmid constructs and to detect the positive clones. In the current study, the positive clones containing L-asparaginase gene were subsequently screened using the colony PCR technique with specific L- asparaginase primers and the produced amplicons were run on the agarose gel electrophoresis that showed a distinct band of size 500 bp. The colony PCR was used in previous studies to detect the positive clones [16, 17, 55–57].

L-asparaginase was then expressed in *E. coli* BL21/pET 22b (+) system. It was reported that *E.coli* BL21 (DE3) is the most commonly utilized laboratory strain for producing recombinant proteins, which was developed by Studier and Moffatt [58]. *E. coli* B strains were developed for protein expression hosts because they lack lon and ompT proteases. *E. coli* BL21 (DE3), a BL21 derivative, is arguably the most often utilized strain for high-level recombinant protein expression. It carries a prophage (DE3), derived from a bacteriophage λ, that is driven by the lacUV5 promoter and bears the T7 RNA polymerase gene also resistant to nalidixic acid [59]. Several studies showed that L-asparaginase can be produced through several vectors and among those systems pET system based on the T7 phage RNA polymerase promoter [18, 60, 61].

Optimization of expression parameters as well as the components can significantly boost protein production to multiple folds. Hence, enzyme production can be made more cost-effective. The role of various parameters including incubation temperature, induction incubation time, IPTG concentration and agitation rate on induction of expression were evaluated. Five different incubation temperatures were tested (20, 25, 30, 37, 40 °C). Expression of L-asparaginase occurred at 20 °C and above, reached its maximum level at 37 °C and decreased slightly at 40 °C. The reduced enzyme expression could be initiated by partial denaturation of the enzyme resulting from changes in metabolic processes. The metabolic activity of microorganisms is slowed by any deviation from the ideal temperature.

The expression of different proteins was time dependent and this was reported in many previous studies [20–22, 25]. The preliminary studies revealed that L-asparaginase expression was a growth linked [62–65]. According to our results there was a gradual rise in the production of the recombinant form of L-asparaginase to show maximum expression level attained at 18 h followed by gradual decrease. Longer incubation times may cause the enzyme to be degraded by proteolytic enzymes, as well as the removal of medium components or the emergence of some inhibitors of the enzyme in the medium, which all result in a drop in the production levels. Additionally, a longer incubation period caused loss of moisture or accumulation of harmful byproducts [66].

Padaria et al., [65] and Jia et al., [67] informed that the amount of expressed protein was improved as the induction time increased as it needed 16 h and 24 h, respectively. Other studies recorded the expression of L-asparaginase at different induction incubation time for 3, 5, 6, 8, 16, and 24 h [18, 62–64, 67–70]. IPTG is used for induction of protein expression [71]. When induction took place in the early stages of exponential development, the bacteria’s metabolic resources were utilized to produce recombinant protein, which accounted for up to 50% of the total protein content of the cell [19]. So, different concentrations extending from 0.1 to 1.2 mM were added at the mid exponential phase and the production of L-asparaginase was measured at each concentration. The expression of L-asparaginase was gradually increased by increasing IPTG concentration then showed the highest expression level when induced at 1 mM compared with the other tested concentrations and at 1.2 mM the expression decreased and this due to the increase of the protein in the insoluble fractions (inclusion bodies) due to the increase in the rate of expression [72]. Similar results were recorded by [18, 62, 69, 70]. While 0.01 mM and 0.5 mM is the optimum IPTG concentration reported by [63, 68], respectively.

By examining the effect of the agitation speed, it was discovered that the agitation speed had a significant impact on the production of enzymes. Tests were conducted using agitation rates of 150, 180, 200, 250 and 270 rpm. At low agitation rates (150 rpm), protein expression was lower than at other rates but at high agitation rates, L-asparaginase expression gradually increased. The highest expression level was reached at 250 rpm. This is due to the agitation rate might have an impact on the medium’s oxygen and nutritional availability, which is a crucial factor in the growth of microorganisms [73]. Increased oxygen content and increased agitation rate aid in nutrients’ mixing, which improves their uptake by microorganisms and leads to growth stimulation and an increase in protein expression [74]. By comparing with other studies, it was found that 200 rpm was the optimum agitation speed [62, 68, 69], while other researchers reported 160 rpm [63] and 180 rpm [70].

Expression was then assessed according to the results obtained for each single factor at a time using RSM experimental model. The optimum range value for the tested factors obtained from the preliminary research were used in the RSM model including 27 experiments with 3 replicates at the center point where the variable to be optimized was the expression of L-asparaginase. The concluded model has a high fit coefficient of R^2^ of 0.98. This refers that the model could present as high as 98.15% of the variability, while only 1.84% of the total variation is not presented by the model. According to the used model, the expected maximum value of L-asparaginase expression was 10.4 IU/ml that can be reached at temperature 37 °C, 250 rpm, and IPTG concentration of 0.83 mM for 17 h. We can conclude that the design could increase the expression of L-asparaginase than the one factor at a time method. The model had adjusted R^2^ of 0.96 meaning that 96% variability in L-asparaginase productivity. The significance of each coefficient was detected by student t-test and *p*-values. These results showed that L-asparaginase production process was significantly affected by all tested factors and the square term of the incubation temperature and post induction incubation time.

According to *p* values, the factor that has the maximum effect on L-asparaginase expression is the linear term of the incubation temperature followed by the square term of incubation temperature as well as linear term of IPTG concentration, induction incubation time, and agitation. These results lead us to a conclusion that these parameters were of maximum effects on L-asparaginase expression and the least change in these will have important effect on its response [75]. Regression equations are represented graphically by 3D response surface plots. Each one of the contour curves depicts an endless array of combinations with the other two maintained at their midpoint values.

The recombinant L- asparaginase enzyme was purified utilizing Ni-NTA columns, the appearance of a definite band on SDS-PAGE assured homogenous purification of recombinant L-asparaginase produced by *E. coli* BL21 (DE3)-pET22b(+)-ASP. The detected molecular mass of the purified L-asparaginase is 17 KDa. Various molecular mass was detected for L-asparaginase from various microorganisms since it was purified from *Enterobacter cloacae*,* Streptomyces brollosae*, *Streptomyces Fradiae*, and *Fusarium foetens* with molecular mass of 52 KDa, 67 KDa, 53 KDa, and 37 KDa, respectively [31, 76, 77]. Additionally a 30 KDa and 25 KDa L-asparaginases were purified from *Bacillus* PG04, and *Bacillus* PG03, respectively [78].

Assessment of the kinetic parameters of the recombinant L-asparaginase enzyme showed that V_max_ was 14.73 IU/ml. The catalytic rate constant was 1.396 S^-1^ while the K_m_ was 2.94 × 10^− 2^ M which is lower than that was found for the purified form of L-asparaginase from *Bacillus licheniformis* of value 0.049 M [79]. The enzyme’s great affinity for L-asparagine as a substrate, as evidenced by the low estimate for the K_m_ received from recombinant *S. maltophilia* L-asparaginase is beneficial for the targeted removal of L-asparagine to target leukemic cells [80]. Previous studies for L-asparaginase’s kinetic parameters had shown various K_m_ estimates from various microorganisms. For example, *Pseudomonas* sp. PCH44 had K_m_ of 0.059 mM [81], *Enterobacter cloacae* had K_m_ of 1.58 × 10^− 3^ M [31] and *Sarocladium strictum* had K_m_ of 9.74 mM [82].

Leukemia and liver cancer are the most significant and common severe types of cancer worldwide [83, 84]. So, we evaluated the in-vitro antitumor activity of the recombinant L- asparaginase produced by *E. coli* BL21 (DE3)-pET22b(+)-ASP in comparison to the marketed L-asparaginase on leukemia cells K-562 and liver cancer cells Hep-G2 using the MTT assay. Half maximum Inhibitory Concentration (IC50) was used to assess the antitumor potential. The results revealed IC50 for the recombinant L-asparaginase of 2.03 IU/ml for K-562 and 1.92 IU/ml for Hep-G2. While the marketed L-asparaginase exhibited higher IC50 of 2.22 IU/ml for K-562 and 2.9 IU/ml for Hep-G2. These results might suggest a higher activity and less systemic toxicity of the recombinant L-asparaginase [85]. Increases in the concentration L-asparaginase were linked to a rise in cytotoxicity with the cancerous cells, suggesting that the cytotoxicity effect was dose-dependent [86]. The anticancer activity of L-asparaginase demonstrated the efficient killing of malignant cell types might be attributed to the deamination of L-asparagine which is a non-essential amino acid led to a diminished asparagine pool [87]. These results indicate that recombinant L-asparaginase produced by *E. coli* BL21 (DE3)-pET22b(+)-ASP was significantly different when compared with the marketed one when studied on Hep-G2 cells with *p*-value < 0.05 while its influence on K-562 was not significantly different.

Numerous investigations had demonstrated the efficiency of L-asparaginase with leukemic cell, as L-asparaginase extracted from *Melioribacter roseus* which had higher IC50 of 3.0 IU/ml [88] than the recombinant L-asparaginase produced by *E. coli* BL21 (DE3)-pET22b(+)-ASP in this study, while the one extracted from *Rhodospirillum rubrum* had IC50 1.8 IU/ml [89] and that from *Halomonas elongate* which had the same IC50 of our enzyme [63]. Also, the IC50 value of the L-asparaginase extracted from *E. cloacae* has been found to be 7.1 IU/ml [31]. Due to the cytotoxic action of the isolated L-asparaginase from *Streptomyces rochei* and *Bacillus velezensis* against Hep-G2 cells, several researchers had confirmed the efficiency of this enzyme on liver cancer Hep-G2 cells [90, 91] and also reported high IC50 4.0 IU from *Streptomyces fradiae* NEAE-82 [76]. In order to ascertain its selective toxicity with cancerous cells and potential cytotoxic influence on healthy normal cells, recombinant L-asparaginase produced by *E. coli* BL21 (DE3)-pET22b(+)-ASP has been evaluated on normal cell MRC-5. The recombinant enzyme didn’t exhibit any cytotoxic effects on the normal cells. This could be explained by the activity of asparagine synthetase, which uses substrate that is provided by other activities when it is depleted [87].

The drug’s immunogenicity is the primary obstacle preventing the usage of foreign proteins in human treatment [40]. It is difficult to develop true immunological tolerance, which requires antigen-specific T-cell-mediated immunosuppression. Changing to an alternative preparation is one way to address this issue momentarily.

EMBOSS antigenic explorer^®^ analysis was used previously to discover L-asparaginases antigenic sites for the *Erwinia chrysanthemi*, *E. coli*, and *S. maltophilia* and the results revealed that they were 16, 18, and 14 antigenic sites, respectively [11, 92].

The antigenic epitopes analysis showed that the antigenicity of L-asparaginase derived from *S. maltophilia* had the lowest level, then the one derived from *Erwinia chrysanthemi*, while *E. coli* had the highest number of antigenic areas. When L-asparaginases which were extracted from *Erwinia* and *E. coli* were utilized medically, partially immune-mediate side effects of the two enzymes were reported [93]. As a result, finding substitute microbial L-asparaginases with fewer immunologically induced side effects becomes important.

The low antigenic epitopes in L-asparaginase of *S. maltophilia* predicted fewer side effects and encouraged additional experimental assessment utilizing an in vivo animal model to illustrate the potential of the enzyme as a promising, novel candidate for medical and pharmaceutical applications.

The antigenicity effect of the recombinant L-asparaginase produced by *E. coli* BL21 (DE3)-pET22b(+)-ASP and the marketed L-asparaginase was compared utilizing male BALB/c mice that were 6–8 weeks old and weighed 20–30 gm. Young mice were selected for the experiment since their immune responses are higher and more sensitive than those of older animals, young mice were selected for the experiment [94, 95]. Besides, their T-cell function and cytokine-releasing ability are excellent [96].

Both recombinant *S. maltophilia* L-asparaginase and marketed L-asparaginase were intraperitoneally administered to the mice. IgG antibody concentrations against both marketed and recombinant L-asparaginase were measured in sera using the ELISA method. The recombinant L-asparaginase produced by *E. coli* BL21 (DE3)-pET22b(+)-ASP was shown to be less immunogenic than the marketed one, with a significant difference in IgG titre levels (*p*-value < 0.0001). Thus, the findings of this study may demonstrate that the recombinant L-asparaginase of *S. maltophilia* is a promising, novel candidate for use as an anticancer drug.

## Conclusion

This study demonstrates the successful cloning and expression of L-asparaginase from *S. maltophilia*. Compared with the commercially available *E. coli* L-asparaginase, the recombinant enzyme produced in *E. coli* BL21 (DE3)-pET22b(+)-ASP exhibited lower immunogenicity and greater selective toxicity, indicating potential anticancer properties. These findings highlight *S. maltophilia* L-asparaginase as a promising novel candidate for development as an anticancer therapeutic agent.

## Data Availability

Please contact author for data request.
